# Direct healthcare costs and their relationships with age at start of drug use and current pattern of use: a cross-sectional study

**DOI:** 10.1590/1516-3180.2020.0115.R1.21102020

**Published:** 2021-12-21

**Authors:** Paula Becker, Denise Razzouk

**Affiliations:** I PhD. Occupational Therapist, Department of Psychiatry, Centro de Economia em Saúde Mental (CESM), Universidade Federal de São Paulo (UNIFESP), São Paulo (SP), Brazil.; II MSc, PhD. Psychiatrist and Affiliated Professor, Department of Psychiatry, Centro de Economia em Saúde Mental (CESM), Universidade Federal de São Paulo (UNIFESP), São Paulo (SP), Brazil.

**Keywords:** Substance-related disorders, Alcohol-related disorders, Crack cocaine, Costs and cost analysis, Community mental health services, Age at initiation, Early start, Late start, Drug dependence costs, Direct costs

## Abstract

**BACKGROUND:**

It is well known that early start of drug use can lead users to psychosocial problems in adulthood, but its relationship with users’ direct healthcare costs has not been well established

**OBJECTIVES:**

To estimate the direct healthcare costs of drug dependency treated at a community mental health service, and to ascertain whether early start of drug use and current drug use pattern may exert influences on these costs.

**DESIGN AND SETTING:**

Retrospective cross-sectional study conducted at a community mental health service in a municipality in the state of São Paulo, Brazil.

**METHODS:**

The relationships between direct healthcare costs from the perspective of the public healthcare system, age at start of drug use and drug use pattern were investigated in a sample of 105 individuals. A gamma-distribution generalized linear model was used to identify the cost drivers of direct costs.

**RESULTS:**

The mean monthly direct healthcare costs per capita for early-start drug users in 2020 were 1,181.31 Brazilian reais (BRL) (274.72 United State dollars (USD) according to purchasing power parity (PPP)) and 1,355.78 BRL (315.29 USD PPP) for late-start users. Early start of drug use predicted greater severity of cannabis use and use of multiple drugs. The highest direct costs were due to drug dependence combined with alcohol abuse, and due to late start of drug use.

**CONCLUSIONS:**

Preventive measures should be prioritized in public policies, in terms of strengthening protective factors before an early start of drug use.

## INTRODUCTION

The prevalence and incidence of drug use in Brazil have increased over recent years, and the age at the start of use has become much lower than in the past.^1^^,^^2^ The Second Brazilian National Survey on Alcohol and Drugs (Levantamento Nacional de Álcool e Drogas, LENAD) showed that among adolescents aged 14 to 17 years, 4.3% were frequent users of cannabis in the past year, 2.3% were frequent users of cocaine, 0.8% frequently used crack and 60% had used alcohol before their 15^th^ birthday.^2^ Additionally, Brazil is ranked as the second largest cocaine market in the world, and national consumption accounts for 20% of the world’s cocaine market.^3^^,^^4^

Early drug use during adolescence is deleterious for the brain maturation process^5^^,^^6^ and has both short and long-term health consequences,^5^^,^^6^^,^^7^^,^^9^ including cognitive impairment,^10^ substance use disorder,^9^ reduced educational and occupational attainment^7^^,^^8^ and engagement in illicit activities.^11^^,^^12^ In this regard, preventive programs have been widely implemented for reducing drug use among adolescents and, consequently, for avoiding economic and social costs.^13^^,^^14^^,^^15^

The great economic impact of substance-related disorders on individuals and society was demonstrated through a study on the burden of diseases in Brazil.^16^ This showed that, among the diseases that contributed most to disability-adjusted life years (DALYs) in this country, disorders relating to use of alcohol and other drugs jumped from third place in 1990 to first in 2016 among men, and from tenth to seventh among women, over the same period. Furthermore, substance-related disorders have been indicated to be one of the costliest health conditions for a healthcare system,^17^^,^^18^^,^^19^ especially regarding hospitalization.^20^

In Brazil, there is a lack of data on the costs according to different drug users’ profiles, especially considering their relationship to age at the start of use. The long-term economic impact of early drug use on the healthcare system needs to be examined. Through this, public healthcare managers can be supported in their decision-making process with regard to allocating the available public healthcare resources more effectively, for prevention and treatment strategies. In this study, we hypothesize that an early start to drug use might be a predictor of higher direct costs for the public healthcare system.

## OBJECTIVES

The aims of this study were to estimate the direct costs due to treatments for individuals dependent on alcohol and other drugs, at a public community mental health service; and to ascertain the potential influences of age at the start of drug use and current drug use pattern on direct healthcare costs. In addition, the potential economic consequences for the public healthcare system were discussed.

## METHODS

### Study design

This was a retrospective cross-sectional study on the relationships between direct healthcare costs and age at the start of drug use and drug use pattern, among individuals undergoing treatment for substance-related disorders at a community mental healthcare service. The cost analysis was conducted from the public healthcare perspective. This study was approved by the Research Ethics Committee of the Federal University of São Paulo (Universidade Federal de São Paulo, UNIFESP), under number 0296, in 2015.

### Setting and participants

The study sample consisted of 105 subjects with a pattern of moderate-to-severe alcohol/drug use who were undergoing treatment at a public community mental health service, the Psychosocial Care Center for Users of Alcohol and Other Drugs (Centro de Atenção Psicossocial para usuários de álcool e outras drogas - CAPS-ad) in the city of Rio Claro, state of São Paulo, Brazil. CAPS-ad is a community-based mental health service that promotes public comprehensive care for people aged 18 years or over with substance-related disorders. It is the reference for substance-related treatment within the public healthcare network in Brazil. This CAPS-ad serves the population of the city of Rio Claro and another four small neighboring municipalities, covering a demographic area with 216,000 inhabitants. The service has a multiprofessional healthcare staff of two psychiatrists, one general practitioner, one nurse, two nursing technicians, two psychologists, two occupational therapists and one social worker.^21^

The inclusion criteria were that the subjects need to be aged 18 years or older, be undergoing treatment at CAPS-ad, be able to understand the interviewer’s questions and meet the criteria for a pattern of moderate or severe drug use with regard to at least one drug, i.e. 11 points or more for alcohol use and 4 points or more for cannabis, alcohol and cocaine/crack use, in accordance with the Alcohol, Smoking and Substance Involvement Screening Test (ASSIST).

#### Early and late-onset drug use

Subjects who began using alcohol, cannabis, cocaine or crack at age 15 years or younger were classified into the “early onset” drug use group. Subjects who started using these drugs at age 16 years or later were classified into the “late onset” drug use group. There is no cutoff age that defines early and late onset of drug use in the literature. It was suggested in some previous studies that this cutoff point could be defined according to the epidemiological data on drug use of the region studied.^9^^,^^22^ In some developed countries, “early onset” drug use has been considered to be use that occurs up to the age of 17 years and “late onset” as use that occurs at the age of 18 years or later.^9^ However, a Brazilian national survey from 2012 showed that the onset of drug use occurred at a much earlier age in this country.^1^^,^^2^

### Data collection

Data on direct health costs were collected using a “bottom-up” approach based on patient-level microdata, through application of the Brazilian version of the Client Socio-Demographic and Service Receipt Inventory (CSSRI),^23^^,^^24^^,^^25^ between March 1, 2015, and August 30, 2017. Information on the number of days in treatment and age at onset of drug use were assessed through a semi-structured questionnaire developed by the research team of this study.

The Alcohol, Smoking and Substance Involvement Screening Test (ASSIST 2.0), which has been validated for use in Brazil,^26^ was applied to evaluate the current alcohol, cannabis and cocaine/crack use pattern. ASSIST consists of eight questions or items that have the aim of investigating the intensity, frequency and problems associated with the use of each substance. The respondents’ answers were classified according to the following categories of use: occasional use (0-3 points), substance abuse (4-26 points for cannabis and cocaine/crack; 11-26 points for alcohol) and possible dependence (27 points or higher).^27^

### Direct healthcare costs

Data on direct costs were collected for the 30 days preceding participation in this study, in relation to the following components:

- *CAPS-ad healthcare staff care* comprised home visits; visits to psychiatrists and general practitioners; and individual and group sessions with occupational therapists, psychologists, social workers and nurses and nurse assistants.
- *Medications* included psychotropic and non-psychotropic medicines.
- *Hospital care* incorporated care received in psychiatric and general hospitals.
- *Outpatient care* included CAPS-III, which has the same CAPS-ad service structure but an around-the-clock service, 24 hours a day and 7 days a week, with crisis support beds for all cases of mental disorders in its coverage area. This also included non-psychiatric medical specialty outpatient services and dental assistance outpatient service.
- *Primary care* included primary care provided by nurses and doctors.
- *Transport* included bus tickets to CAPS-ad, emergency mobile medical care (Serviço de Atendimento Móvel de Urgência, SAMU) and inter-municipal transportation for treatment at CAPS-ad.

Unit costs were available for the year 2015. These were then adjusted for inflation up to the year 2018, in accordance with the general market price index (IGP-M), a Brazilian inflation rate index measured by the Getúlio Vargas Foundation (Fundação Getúlio Vargas, FGV).^28^ Costs in Brazilian reais (BRL) were also converted to United States dollars (USD) using purchasing power parity (PPP) exchange rates.^29^

The unit cost values were calculated by means of a top-down approach, in accordance with municipal accounting data provided by the local public healthcare manager.^30^ For situations in which these data were not available, the current scientific literature was consulted.^31^^,^^32^

Unit costs for medications were estimated from information provided by the municipal government regarding the prices paid for these medicines in the year 2015. For some medicines used by the subjects, the purchase prices were not available from the municipal government. In such situations, the medication prices database,^33^ a Brazilian database on prices paid by the public healthcare sector for purchases of medicines, was consulted.

### Data analysis

Initially, descriptive analysis was conducted. This was followed by an analysis on associations between variables and early and late onset of drug use. Associations between nominal variables were verified using the chi-square test or, in cases of small samples, Fisher’s exact test. Student’s t test was used to compare mean costs and the nonparametric Mann-Whitney test was used to compare numerical variables of non-normal distribution.

Inferential analysis was then conducted, in which “direct cost” was defined as the dependent variable in a gamma-distribution generalized linear model (GD-GLM) with a log binding function and marginal gamma distribution.^34^ This model was chosen because of the nature of the dependent variable which was numerical, with non-negative values and asymmetry. The reasonableness of choosing this distribution was verified using Anscombe residuals.^34^

The GD-GLM had two sequential stages of analysis: univariate and multivariate. For the univariate analysis, variables that demonstrated significant associations (a significance level of 5% or P ≤ 0.05) with the age of onset of drug use and those that we intended to investigate as possible direct cost predictors were selected. Predictive variables that showed associations with the dependent variable at a significance level of 20% in the univariate analysis, except for the current age and time of treatment (control variables), were selected for the multivariate models.

The choice of a significance level of 20% came from the relationship between sample size and the number of predictor variables analyzed in the univariate regression model. In other words, it was considered that variables showing significance of up to 20% in the univariate model could be significant at 5% in the final multivariate model. Thus, no significant predictive variable would be disregarded for the final multivariate regression model. For the predictive variables present in numerical and categorical forms that were both significant in the univariate model, the form in which the association with the dependent variable was more significant was selected. Subsequently, the variables that did not present significance at the 5% level were excluded one by one, in order of significance, using the backward method. The analyses were performed using the STATA 12 (StataCorp, Texas, 2011)^35^ statistical package.

## RESULTS

Totals of 59 early-onset substance users (56.2%) and 46 late-onset substance users (43.8%) composed the study sample (n = 105). The mean ages at onset of alcohol, cannabis, cocaine and crack use were, respectively, 15.2 years (standard deviation, SD = 5.7), 15.6 years (SD = 5.6), 20.2 years (SD = 8.6) and 23.9 years (SD = 12).

Table 1 shows the sociodemographic profile of the sample according to early or late onset of drug use. The mean age of the entire sample was 42.7 years (SD = 11.0), and there was a significant difference (P = 0.01) between the ages of the early-onset group (40.5 years; SD = 11.0) and the late-onset group (45.6 years; SD = 9.4). On average, early-exposed users were five years younger than the late-exposed users. The mean length of time spent undergoing the current treatment at CAPS-ad was 46.4 days overall (SD = 87.8). For the early-onset group, this number was 42.2 days (SD = 83.7) and for the late-onset group it was 51.7 days (SD = 93.6) (P = 0.58).

**Table 1.** Sociodemographic profile of the sample according to early or late onset of drug use

	Total n (%)	Early onset (n = 59) n (%)	Late onset (n = 46) n (%)	P
Gender (male)	86 (81.9)	49 (83.1)	37 (80.4)	0.73
Marital status				
Single	52 (49.5)	35 (59.3)	17 (37.0)	0.07
Married	32 (30.5)	13 (22.0)	19 (41.3)	
Divorced	18 (17.1)	9 (15.3)	9 (19.6)	
Widower	3 (2.9)	2 (3.4)	1 (2.2)	
Religion				
Catholic	38 (36.2)	21 (35.6)	17 (37.0)	0.50
Protestant	47 (44.8)	24 (40.7)	23 (50.0)	
Atheist	12 (11.4)	9 (15.3)	3 (6.5)	
Other	8 (7.6)	5 (8.5)	3 (6.5)	
Educational level				
Illiterate	3 (2.9)	1 (1.7)	2 (4.3)	0.15
Incomplete elementary school	42 (40.0)	23 (39.0)	19 (41.3)	
Completed elementary school	26 (24.8)	15 (25.4)	11 (23.9)	
Incomplete high school	10 (9.5)	9 (15.3)	1 (2.2)	
Completed high school	23 (21.9)	11 (18.6)	12 (26.1)	
Postgraduate	1 (1.0)	0 (0.0)	1 (2.2)	
Occupation				
Formally employed	26 (24.8)	16 (27.1)	10 (21.7)	0.65
Sick leave	3 (2.9)	1 (1.7)	2 (4.3)	
Retired	7 (6.7)	3 (5.1)	4 (8.7)	
Informal job	11 (10.5)	7 (11.9)	4 (8.7)	
Unemployed	58 (55.2)	32 (54.2)	26 (56.5)	
Not the first treatment attempt at CAPS-ad	55 (52.4)	32 (54.2)	23 (50.0)	0.66

Chi-square test or Fisher’s test, P ≤ 0.05. CAPS-ad = Psychosocial Care Center for Users of Alcohol and Other Drugs.

Table 2 presents data on past and current drug use patterns, as measured through ASSIST, according to early or late onset of drug use. There were significant differences between the early and late onset groups regarding the *second drug of experimentation* and *current cannabis use pattern*. More than half (54.3%) of the subjects with late-onset drug use did not try a second drug or further drugs, compared with 28.8% of the early-onset group (P = 0.02). This latter group had a higher number of subjects who met the criteria for abuse and possible dependence on cannabis, compared with the group of late-onset users (P = 0.04).

**Table 2.** Lifetime and current drug use pattern (ASSIST) according to early or late onset of drug use

	Total (n = 105) n (%)	Early onset (n = 59) n (%)	Late onset (n = 46) n (%)	P
Lifetime drug use				
First drug of experimentation				
Alcohol	63 (62.4)	38 (64.4)	25 (59.5)	0.24
Cannabis	14 (13.9)	9 (15.3)	5 (11.9)	
Cocaine/crack	11 (10.9)	8 (13.6)	3 (7.1)	
Alcohol and cannabis	5 (5.0)	1 (1.7)	4 (9.5)	
Multiple drugs	8 (7.9)	3 (5.1)	5 (11.9)	
Second drug of experimentation				
Alcohol	10 (9.9)	6 (10.2)	4 (9.5)	0.00
Cannabis	20 (19.8)	11 (18.6)	9 (21.4)	
Cocaine/crack	16 (15.8)	13 (22.0)^A^	3 (7.1)^B^	
Multiple drugs	13 (12.9)	12 (20.3) ^A^	1 (2.4) ^B^	
None	42 (41.6)	17 (28.8)^A^	25 (59.5)^B^	
Age at first use: mean (SD)				
Alcohol^1^	15.2 (5.7)	13.2 (2.5)	18.1 (7.5)	< 0.00^b^
Cannabis^2^	15.6 (5.6)	14.5 (4.4)	17.6 (7.2)	< 0.00^c^
Cocaine^3^	20.2 (8.6)	17.6 (5.0)	25.0 (11.6)	0.00^c^
Crack^4^	23.9 (12.0)	23.3 (11.0)	25.0 (14.3)	0.66^b^
Current drug use pattern				
ASSIST − alcohol				
Occasional use	24 (22.9)	11 (23.9)	13 (22.0)	0.90
Abusive use	32 (30.5)	13 (28.3)	19 (32.2)	
Possible dependence	49 (46.7)	22 (47.8)	27 (45.8)	
ASSIST - cannabis				
Occasional use	80 (76.2)	40 (87.0)	40 (67.8)	0.04^a^
Abusive use	23 (21.9)	6 (13.0)	17 (28.8)	
Possible dependence	2 (1.9)	0 (0.0)	2 (3.4)	
ASSIST - cocaine/crack				
Occasional use	59 (56.2)	28 (60.9)	31 (52.5)	0.39
Abusive use	25 (23.8)	8 (17.4)	17 (28.8)	
Possible dependence	21 (20.0)	10 (21.7)	11 (18.6)	

Chi-square test or Fisher’s test(^a^), and Student’s t(^b^) or Mann-Whitney(^c^), P ≤ 0.05; (A) and (B) show different percentages between early and late-onset groups; ^1^Only for subjects who had used alcohol; ^2^Only for subjects who had used cannabis; ^3^Only for subjects who had used inhaled cocaine; ^4^Only for subjects who had used crack; ASSIST = Alcohol, Smoking and Substance Involvement Screening Test; SD = standard deviation.

Table 3 describes the subjects’ consumption of healthcare network resources. On average, the late-onset group more often used *group sessions with nurse* (P = 0.04) *and psychologist* (P = 0.03), *nurse routine individual care sessions* (P = 0.00) and *visits to a general practitioner* (P = 0.04). These results are reflected in the direct healthcare costs per capita, shown in Table 4. The late-onset group showed higher mean monthly costs for *visits to general practitioner* (P = 0.04), *group sessions with nurse* (P = 0.04), *group sessions with psychologist* (P = 0.01), *nurse routine individual care sessions* (P = 0.00) and *bus ticket to CAPS-ad* (P = 0.04), in comparison with the early-onset group.

**Table 3.** Consumption of healthcare network resources over the last 30 days, in 2015

	Total (n = 105)			Early onset (n = 59)			Late onset (n = 46)			P
	n	Mean (SD)	Minimum-Maximum	n	Mean (SD)	Minimum-Maximum	n	Mean (SD)	Minimum-Maximum	
CAPS-ad										
Home visits	4	1.0 (0.0)	1-1	1	1.0 (0.0)	1-1	3	1.0 (0.0)	1-1	-
Visits to psychiatrist	85	1.2 (0.5)	1-4	44	1.3 (0.6)	1-4	41	1.1 (0.4)	1-3	0.19
Visits to general practitioner	28	1.1 (0.4)	1-3	13	1.0 (0.0)	1-1	15	1.3 (0.6)	1-3	0.04
Group session with nurse	62	3.9 (5.3)	1-40	35	3.8 (6.7)	1-40	27	4.1 (3.0)	1-12	0.04
Nurse routine individual care session	99	12.6 (8.4)	1-42	57	10.3 (7.7)	1-30	42	15.8 (8.3)	2-42	0.00
Group session with psychologist	86	5.7 (4.7)	1-30	47	4.6 (3.1)	1-12	39	7.1 (5.8)	1-30	0.03
Individual session with psychologist	46	3.7 (4.0)	1-20	26	4.0 (5.1)	1-20	20	3.3 (1.6)	1-8	0.26
Group session with social worker	75	4.2 (4.6)	1-24	44	4.0 (4.5)	1-24	31	4.4 (4.7)	1-24	0.36
Individual session with social worker	47	3.1 (3.10)	1-16	26	2.4 (2.3)	1-12	21	3.9 (3.8)	1-16	0.14
Group session with occupational therapist	93	5.48 (4.6)	1-24	50	5.2 (5.0)	1-24	43	5.7 (4.2)	1-18	0.30
Individual session with occupational therapist	24	2.5 (1.7)	1-8	12	2.5 (1.9)	1-8	12	2.5 (1.6)	1-6	0.95
Hospital care										
Psychiatric hospital (days)	5	10.8 (6.5)	4-20	2	17.5 (3.5)	15-20	3	6.3 (2.0)	4-8	0.08
General hospital (visits to ER)	30	1.7 (1.4)	1-6	15	1.4 (0.9)	1-4	15	2.0 (1.8)	1-6	0.89
Outpatient care										
CAPS-III	5	1.0 (0.0)	1-1	2	1.0 (0.0)	1-1	3	1.0 (0.0)	1-1	1.00
Non-psychiatric medical specialties outpatient service	7	1.5 (0.7)	1-3	4	1.5 (0.5)	1-2	3	1.6 (1.1)	1-3	1.00
Dental assistance outpatient service	6	2.1 (1.1)	1-4	2	1.0 (0.0)	1-1	4	2.7 (0.9)	2-4	0.57
Primary care										
Nurse primary care	12	1.2 (0.8)	1-4	7	1.4 (1.1)	1-4	5	1.0 (0.0)	1-1	0.39
Doctor primary care	17	1.9 (1.1)	1-5	7	2.1 (1.4)	1-5	10	1.8 (0.9)	1-3	0.75
Transportation										
Bus ticket to CAPS-ad	55	2.0 (0.9)	1-9	32	1.9 (0.2)	1-2	23	2.3 (1.4)	2-9	0.91
Emergency mobile medical care (per call)	12	1.2 (0.8)	1-4	8	1.0 (0.0)	1-1	4	1.7 (1.5)	1-4	0.15
Intermunicipal transportation for treatment at CAPS-ad (per trip)	10	11.9 (12.7)	2-40	7	10.5 (13.4)	2-40	3	15.0 (13.0)	7-30	0.35

Mann-Whitney test or Student’s t test, P ≤ 0.05; SD = standard deviation; CAPS-ad = Psychosocial Care Center for Users of Alcohol and Other Drugs.

**Table 4.** Direct healthcare costs per capita over the last 30 days, in 2015

	Unit costs description	Cost per unit − BRL	Total (n = 105)		Early onset (n = 59)		Late onset (n = 46)		P
			Mean (SD) − BRL	Minimum- Maximum − BRL	Mean (SD) - BRL	Minimum- Maximum − BRL	Mean (SD) − BRL	Minimum- Maximum − BRL	
TOTAL DIRECT COSTS			863.8 (1,396.48)	33.20-8,338.30	778.74 (1,439.14)	33.20-8,338.30	972.91 (1,347.57)	40.35-6,303.13	0.48
CAPS-ad									
Home Visits	One 60-minute home visit by a nurse assistant and a higher-education healthcare professional	35.91	40.45 (5.24)	35.91-44.98	35.91 (0.00)	35.91-35.91	41.96 (5.24)	35.91-44.98	-
Visits to psychiatrist	One individual visit of 30 min	28.16	34.79 (16.05)	28.16-112.64	37.12 (18.99)	28.16-112.64	32.28 (11.88)	28.16-84.48	0.16
Visits to general practitioner	One individual visit of 30 min	28.16	33.19 (13.39)	28.16-84.48	28.16 (0.00)	28.16-28.16	37.55 (17.38)	28.16-84.48	0.04
Group session with nurse	One 90-min group session, with an average of 10 patients	2.97*	11.78 (16.03)	2.97-118.80	11.29 (19.99)	2.97-118.8	12.43 (8.95)	2.97-35.64	0.04
Nurse routine individual care session	One individual session of 15 min to evaluate the patient’s general state of health	3.35	42.5 (28.25)	3.35-140.70	34.62 (25.86)	3.35-100.5	53.20 (28.11)	6.70-140.70	0.00
Group session with psychologist	One 90-min group session, with an average of 10 patients	2.80*	16.15 (13.24)	2.80-84.00	12.87 (8.94)	2.80-33.6	20.10 (16.31)	2.80-84.00	0.01
Individual session with psychologist	One individual session of 60 min	18.73	69.22 (75.53)	18.73-374.6	74.92 (97.25)	18.73-374.6	61.81 (31.05)	18.73-149.84	0.26
Group session with social worker	One 90-min group session, with an average of 10 patients	3.96*	16.63 (18.32)	3.96-95.04	15.93 (18.13)	3.96-95.04	17.63 (18.85)	3.96-95.04	0.69
Individual session with social worker	One individual session of 60 min	26.44	82.13 (83.83)	26.44-423.04	64.07 (62.2)	26.44-317.28	104.5 (101.88)	26.44-423.04	0.14
Group session with occupational therapist	One 90-min group session, with an average of 10 patients	3.74*	20.51 (17.43)	3.74-89.76	19.67 (18.9)	3.74-89.76	21.48 (15.72)	3.74-67.32	0.62
Individual session with occupational therapist	One individual session of 60 min	24.98	63.49 (44.18)	24.98-199.84	64.53 (49.34)	24.98-199.84	62.45 (40.56)	24.98-149.88	0.95
MEDICATIONS		**	15.51 (15.44)	1.68-83.25	14.21 (12.7)	1.68-52.35	17.13 (18.33)	1.68-83.25	0.48
Psychotropic	Medication price database and municipal data	**	10.97 (13.04)	0.84-79.44	9.72 (10.65)	0.84-52.35	12.49 (15.47)	0.84-79.44	0.99
Non-psychotropic		**	8.09 (13.28)	0.48-80.76	9.18 (11.40)	1.68-40.68	7.04 (15.01)	0.48-80.76	0.06
Hospital care									
Psychiatric hospital	One bed per day of hospitalization^31^	369.08	3,995.81 (2,424.25)	1,476.32-7,414.31	6,480.60 (1,320.47)	5,546.88-7,414.31	2,339.29 (769.34)	1,476.32-2,953.39	0.08
General hospital	One visit to emergency room (average cost of emergency care at a general medium-sized general hospital emergency room)^30^	436.23	1,036.10 (916.87)	436.26-4,345.74	898.23 (663.79)	436.26-2,810.4	1,173.97 (1.122.)	436.26-4,345.74	0.42
Outpatient care									
CAPS-III	12-hour night bed (psychiatrist and nursing staff cost, and non-medical direct costs) - municipal data	388.46	86.69 (168.69)	11.25-388.46	199.86 (266.73)	11.25-388.46	11.25 (0.00)	11.25-11.25	0.22
Non-psychiatric medical specialties outpatient service	One appointment (service total average cost in 2015 divided by total number of appointments) - municipal data	115.44	181.41 (90.83)	115.44-346.32	173.16 (66.65)	115.44-230.88	192.40 (133.30)	115.44-346.32	1.00
Dental assistance outpatient service	One appointment (service total average cost in 2015 divided by total number of appointments) - municipal data	95.31	206.51 (111.42)	95.31-381.24	95.31 (0.00)	95.31-95.31	262.10 (91.25)	190.62-381.24	0.05
Primary care									
Nurse primary care	One individual session of 60 min with nurse	18.71	39.13 (20.36)	18.71-74.84	42.94 (20.86)	18.71-74.84	33.8 (20.66)	18.71-56.43	0.43
Doctor primary care	One individual visit of 30 min	70.07	136.02 (80.16)	70.07-350.35	150.15 (102.57)	70.07-350.35	126.13 (64.39)	70.07-210.21	0.75
Transportation									
Ticket for transportation to CAPS-ad	One voucher	3.30	113.76 (100.46)	9.90-594.00	94.67 (84.70)	9.90-396.00	135.05 (113.45)	13.20-594.00	0.04
Emergency mobile medical care	One emergency call	195.28	244.10 (169.12)	195.28-781.12	195.28 (0.00)	195.28-195.28	341.74 (292.92)	195.28-781.12	0.15
Intermunicipal transportation for treatment at CAPS-ad	One round-trip (average cost considering driver, fuel and toll costs divided by the average number of patients transported)	14.90	86.39 (90.73)	14.90-286.40	76.77 (95.8)	14.90-286.40	108.85 (91.83)	52.15-214.80	0.35

Mann-Whitney test or Student’s t test, P ≤ 0.05; *Cost of one group session per patient; **Unit cost varied according to each medication; SD = standard deviation; BRL = Brazilian real; CAPS-ad = Psychosocial Care Center for Users of Alcohol and Other Drugs; CAPS-III = has the same CAPS-ad service structure but an around-the-clock service, 24 hours a day and 7- days a week, with crisis support beds for all cases of mental disorders in the coverage area.

The mean monthly per capita direct cost adjusted for inflation in 2020 was BRL 1,181.31 (USD 274.72 PPP) for the early-onset drug use group and BRL 1,355.78 (USD 315.29 PPP) for the late-onset drug use group. The mean CAPS-ad treatment cost (including healthcare staff assistance, home visits and use of both psychotropic and non-psychotropic medications) was BRL 266.27 in 2015 (BRL 380.66, i.e. USD 88.52 PPP, in 2020) and accounted for 30.8% of per capita total direct cost.

Table 5 presents the GD-GLM univariate analysis results. Predictive variables for which the associations with direct healthcare costs were significant at the 20% level at this stage were selected for multivariate analysis.

**Table 5.** Results from univariate gamma regression models for direct costs

	Average ratio (95% CI)	P
Gender (female) (ref. = male)	0.939 (0.419-2.108)	0.88
Educational level (ref. = illiterate/incomplete elementary school)		
Completed elementary school/Incomplete high school	1.031 (0.525-2.026)	0.93
Completed high school or more	0.672 (0.313-1.441)	0.30
School dropout	1.263 (0.643-2.482)	0.49
Days in current treatment at CAPS-ad	0.998 (0.993-1.003)	0.42
Age (years)	1.007 (0.974-1.041)	0.69
Age at onset of drug use		
First drug	1.015 (0.998-1.033)	0.08
Second drug	1.014 (1.000-1.028)	0.04
Alcohol	0.969 (0.927-1.014)	0.17
Cannabis	1.000 (0.967-1.033)	0.99
Cocaine	1.006 (0.982-1.032)	0.62
Crack	1.009 (0.986-1.032)	0.45
Duration of drug use (years)		
Alcohol	0.994 (0.973-1.017)	0.62
Cannabis	0.991 (0.968-1.014)	0.44
Cocaine	0.997 (0.969-1.026)	0.83
Crack	1.012 (0.974-1.050)	0.54
ASSIST (highest score among all drugs)	0.996 (0.957-1.037)	0.84
ASSIST (total numerical score)		
Alcohol	1.017 (0.987-1.048)	0.26
Cannabis	0.992 (0.952-1.033)	0.69
Cocaine/crack	1.000 (0.977-1.024)	0.99
ASSIST - alcohol (ref. = occasional use)		
Abusive use	4.771 (2.445-9.313)	< 0.00
Possible dependence	2.355 (1.271-4.365)	0.00
ASSIST - cannabis (ref. = occasional use)		
Abusive use	0.866 (0.409-1.834)	0.70
Possible dependence	0.329 (0.034-3.180)	0.33
ASSIST - cocaine/crack (ref. = occasional use)		
Abusive use	1.596 (0.768-3.316)	0.21
Possible dependence	0.892 (0.409-1.942)	0.77

P ≤ 0.05; CI = confidence interval; ref. = reference; CAPS-ad = Psychosocial Care Center for Users of Alcohol and Other Drugs; ASSIST = Alcohol, Smoking and Substance Involvement Screening Test.

Table 6 presents the results relating to the multivariate GD-GLM. In the final model, the predictive variables *age of onset of first drug use* (P = 0.034), *ASSIST alcohol-abusive use* (P < 0.001) and *ASSIST alcohol-possible dependence* (P = 0.049) remained significant. These results showed that for each year later at which the first drug experimentation occurred there was a 1.1% increase in total direct cost.

**Table 6.** Results from initial and final multivariate gamma regression models for direct costs

	Initial model		Adjusted final model	
	average ratio (95% CI)	P	average ratio (95% CI)	P
Days in current treatment at CAPS-ad	1.000 (0.996-1.004)	0.899	1.000 (0.996-1.004)	0.90
Age (years)	1.006 (0.978-1.036)	0.673	1.005 (0.979-1.031)	0.72
Age at onset of drug use				
First drug	1.011 (0.981-1.043)	0.472	1.015 (1.001-1.029)	0.03
Second drug	1.003 (0.978-1.029)	0.821	-	-
Alcohol	0.996 (0.953-1.042)	0.876	-	-
ASSIST − alcohol (ref. = occasional use)				
Abusive use	4.288 (2.102-8.749)	< 0.001	4.381 (2.210-8.688)	< 0.00
Possible dependence	1.996 (0.976-4.082)	0.058	2.023 (1.002-4.084)	0.04

CI = confidence interval; CAPS-ad = Psychosocial Care Center for Users of Alcohol and Other Drugs; ASSIST = Alcohol, Smoking and Substance Involvement Screening Test; ref. = reference.

In addition, treatment for drug dependents who were also alcohol abusers was 4.4 times more expensive than for dependents who did not use alcohol, and treatment for alcohol-dependent users was twice as expensive as for those who did not use alcohol. Drug dependents who were also alcohol abusers had a higher monthly average direct cost (BRL 2,247.53, i.e. USD 522.68 PPP, *per capita* in 2020) than that of drug dependents who only made occasional use of alcohol (BRL 471.06, i.e. USD 109.54 PPP, *per capita* in 2020) (P = 0.002), as can be seen in Table 7.

**Table 7.** Per capita direct costs according to ASSIST results for alcohol, cannabis and cocaine/crack, in 2015 - Brazilian reais (BRL)

Direct costs	N	Mean	Standard deviation	Minimum	Maximum	1^st^ quartile	Median	3^rd^ quartile	P
	105	863.80	1,396.48	33.20	8,338.30	171.91	452.57	780.52	
Alcohol									
Occasional use	24	310.53^B^	228.63	33.20	807.49	77.06	288.12	498.02	0.00
Abusive use	32	1,481.62^A^	2,072.98	37.79	8,338.30	326.82	638.88	1,440.60	
Possible dependence	49	731.32	999.79	47.18	5,268.34	168.28	455.64	802.73	
Cannabis									
Occasional use	80	901.71	1,418.34	33.20	8,338.30	183.24	460.73	774.44	0.57
Abusive use	23	781.26	1,396.99	40.17	6,824.33	103.60	354.12	807.49	
Possible dependence	2	296.51	210.15	147.91	445.10	-	-	-	
Cocaine/crack									
Occasional use	59	771.09	1.063.24	47.18	5,268.34	173.35	438.77	800.40	0.38
Abusive use	25	1,230.72	2,015.31	33.20	8,338.30	162.32	536.89	1,228.38	
Possible dependence	21	687.48	1.327.56	37.79	6,303.13	135.48	312.95	621.05	

Kruskal-Wallis test, P ≤ 0.05; (-) not presented due to the small number of cases; (A) and (B) presented different means according to Dunn-Bonferroni multiple comparisons.

## DISCUSSION

The direct costs were higher for the subjects who met the criteria for both drug-related dependence and alcohol abuse, and were also higher among those in the late-onset group. One potential explanation for the higher costs among late-onset drug users may be that, as demonstrated by previous studies,^9^ these users’ profiles show that they had better adherence to the proposed treatments. This may imply better treatment outcomes and higher direct costs to the public healthcare system, in comparison with those of early-onset drug users.

However, the sample selection bias, small sample size, retrospective study design and low representativeness of all alcohol and drug users’ profiles may also have influenced this result. Considering the low adherence to treatment among early-onset drug users, we hypothesize that if they developed a severe drug use pattern earlier than the late-onset group, the early-onset drug users would be unlikely to be found in the community mental health center. Therefore, the direct costs of early-onset drug users may have been underestimated because they may have been accessing types of treatment that were more complex and more costly (i.e. hospitalizations), and because the present study did not consider costs relating to mortality.

This hypothesis can be discussed in the light of results from previous studies. A Brazilian study demonstrated that crack and cocaine users aged 25 years or over fitted a drug user profile that was quite prevalent and recurrent in general hospital emergency rooms in São Paulo.^36^ A 30-year prospective study conducted in New Zealand found that substance dependence, failure to obtain educational qualifications and criminal convictions in adulthood were predicted by early exposure to drugs (up to age 15 years).^37^ Andreuccetti et al.^38^ found that 37% of the victims of violent, sudden or unexpected deaths in the city of São Paulo were younger than 30 years of age; 55.3% had ingested alcohol (the most prevalent drug) or had used other drugs (cocaine, cannabis or sedatives and anxiolytics, in decreasing prevalence) before they died; and 15.9% had some form of criminal history. Among this last group, the rate of use of drugs other than alcohol and the rate of use of multiple drugs were higher than they were among victims who had no criminal history.

Although the early onset of drug use did not predict higher direct costs in the way in which we had originally hypothesized this, it did predict greater severity of cannabis and multiple drug use in adulthood. These data corroborate a 2017 Brazilian study conducted by Castaldelli-Maia et al.^39^ that showed that there is an ongoing change in the role that cannabis plays in the culture of drug experimentation among Brazilian adolescents. Moreover, these data indicate that, as is also occurring in other countries like Spain,^40^ the age at which cannabis experimentation starts is becoming similar to the ages at which alcohol and tobacco use start. This same study also showed that cannabis use acted as a predictor of alcohol use and had significant relationships with subsequent use of cocaine, prescription opioids and tranquillizers.

Therefore, these data can inform policymakers and society about the risks of early-onset cannabis use, considering the important role that early-onset use of this drug could be playing in predicting subsequent abuse of and dependence upon multiple drugs. These data also reinforce the notion that preventive measures should be prioritized in substance-related national policies in terms of strengthening protective factors before early-onset drug use might occur, and in the interests of preventing further severe and multiple drug use.

In 2013, three public preventive programs targeting drug use were implemented in Brazil.^41^ However, no official data exist in relation to the implementation costs of these programs; moreover, the effectiveness of only one of these programs, the *#*TamoJunto program, has been evaluated. Sanchez et al.^42^ investigated this program through a randomized clinical trial (RCT).

The *#*TamoJunto program, a Brazilian adaptation of a European program called Unplugged,^43^ was implemented at high schools, focusing on adolescents aged 10 to 14 years. Unlike the European program, in which exposure to Unplugged was associated with significantly lower prevalence of daily use of cigarettes, episodes of drunkenness and use of cannabis over the past thirty days,^44^ the Brazilian version promoted a protective effect regarding first inhalant use, had no effects on the prevalence of past-month drug use and showed increased relative risk of first alcohol use, i.e. a potential iatrogenic effect. In addition, the RCT demonstrated that the program had no effect on students’ beliefs about drug use but found that those who originally had more negative beliefs about drug use had lower drug consumption during the follow-up than those who had positive beliefs.^45^ These results indicate that there is a need for further studies that consider Brazilian cultural factors, in order to implement preventive public policies regarding drug use among youths in this country. Thus, the results led the federal government to reconsider continuation of the #TamoJunto program expansion as a public drug prevention policy.^41^

Despite Brazil’s initial attempts to implement drug use prevention programs, national alcohol and drug policies have mainly been directed towards adults with substance-related disorders, and with a focus on resource allocation to hospitalization and tertiary-level treatment.^46^^,^^47^ This is due particularly to the current austerity policy and the resource allocation constraints that Brazil has been facing as a result of the economic crisis over recent years. In 2018, for instance, the Brazilian federal government invested BRL 90 million, comprising BRL 40 million from the Ministry of Justice, BRL 10 million from the Ministry of Social Development and BRL 40 million from the Ministry of Health, in private clinics that focus on inpatient treatment for drug dependence. However, such treatments have not been proven to be effective or cost-effective for treating people with substance-related disorders.^48^ This scenario underscores the importance of economic evidence for planning drug and alcohol policies.

 In terms of the economic impact on the public healthcare system, the mean monthly direct costs per capita in our sample were almost 4.4 times greater than the mean per capita public healthcare expenditures in Brazil in 2015, while the costs for those who were both drug dependent and alcohol abusers were 7.5 times greater than national per capita healthcare expenditure in that year, according to data from the Organization for Economic Cooperation and Development.^49^ According to another national survey developed by the Brazilian Federal Council of Medicine,^50^ the mean annual per capita healthcare expenditure in São Paulo in 2017 was BRL 656.91, representing only 3.2% of the mean monthly per capita cost of a drug dependent who is also an alcohol abuser (BRL 1,728.43 or USD 519.00 in 2017).

The current drug use situation in Brazil has alerted healthcare workers and government officials to the need to estimate its economic impact on the Brazilian public healthcare system in order to develop specific public policies, especially focused on prevention, targeting the highest risk groups. Public policies oriented toward preventing early-onset drug use among adolescents may reduce the economic impact that substance-related disorders have on the public healthcare system. These may also help adolescents avoid both developing dependence upon multiple drugs and having their consequences in adulthood.

There are several limitations to this study. Two related limitations comprised the small sample size and low representativeness of all the alcohol and drug users’ profiles. This indicates that caution is needed in making generalizations. Another limitation was the retrospective study design, which did not permit analysis of possible cost variations according to each user’s profile from his or her age at the onset of drug use to the age at the time of participation in this study. Lastly, there was some uncertainty regarding inaccuracies of cost estimations, given the large territorial extent of Brazil and regional differences in values aggregated to the components of the costs considered.

## CONCLUSIONS

Our results are useful for alerting policymakers towards addressing national preventive policies against drug use, for the young population. Preventive measures should be prioritized within national alcohol and drug policies, in order to strengthen protective factors before early onset of drug use, especially regarding alcohol and cannabis, and to avert further severe and multiple drug use. Therefore, our findings suggest that there is a need to conduct further prospective studies on adolescents’ drug use, their pathways through the healthcare system, the costs of their drug use and the social outcomes among these individuals.
